# Femoral defects in revision hip arthroplasty: a therapy-oriented classification

**DOI:** 10.1007/s00402-021-04201-7

**Published:** 2021-10-12

**Authors:** Max Jaenisch, Hendrik Kohlhof, Adnan Kasapovic, Martin Gathen, Thomas Martin Randau, Koroush Kabir, Philip Peter Roessler, Geert Pagenstert, Dieter Christian Wirtz

**Affiliations:** 1 Department of Orthopedics and Trauma Surgery, University Hospital Bonn, Venusberg Campus 1, 53127 Bonn, Germany; 2 CLARAHOF Clinic of Orthopaedic Surgery, University of Basel, Clarahofweg 19a, 4058 Basel, Switzerland

**Keywords:** Femoral, Bone defect, Hip, Arthroplasty, Revision

## Abstract

**Introduction:**

The complex field of femoral defects in revision hip arthroplasty displays a lack of standardized, intuitive pre- and intraoperative assessment. To address this issue, the femoral defect classification (FDC) is introduced to offer a reliable, reproducible and an intuitive classification system with a clear therapeutic guideline.

**Materials and methods:**

The FDC is based on the integrity of the main femoral segments which determine function and structural support. It focuses on the femoral neck, the metaphysis consisting of the greater and lesser trochanter, and the femoral diaphysis. The four main categories determine the location of the defect while subcategories a, b and c are being used to classify the extent of damage in each location. In total, 218 preoperative radiographs were retrospectively graded according to FDC and compared to intraoperatively encountered bone defects. To account for inter-rater and intra-rater agreement, 5 different observers evaluated 80 randomized cases at different points in time.

**Results:**

A Cohens kappa of 0.832 ± 0.028 could be evaluated, accounting for excellent agreement between preoperative radiographs and intraoperative findings. To account for inter-rater reliability, 80 patients have been evaluated by 5 different observers. Testing for inter-rater reliability, a Fleiss Kappa of 0.688 could be evaluated falling into the good agreement range. When testing for intra-rater reliability, Cohens Kappa of each of the 5 raters has been analyzed and the mean was evaluated at 0.856 accounting for excellent agreement.

**Conclusion:**

The FDC is a reliable and reproducible classification system. It combines intuitive use and structured design and allows for consistent preoperative planning and intraoperative guidance. A therapeutic algorithm has been created according to current literature and expert opinion. Due to the combination of the FDC with the recently introduced Acetabular Defect Classification (ADC) a structured approach to the entire field of hip revision arthroplasty is now available.

## Introduction

Modern total hip arthroplasty continues to be on the rise and implantation rates are predicted to keep increasing all over the developed world [1]. In the near future, orthopedic surgeons will be faced with more and more complex cases of bone loss while revision and re-revision surgeries are piling up. Acetabular defects have been sufficiently described and organized with the recent addition of the acetabular defect classification (ADC) published by this group [2]. However, for femoral defects a lack of standardized, intuitive pre and intraoperative assessment is still being experienced.

Successful treatment of femoral bone defects focuses on the reconstruction of the physiological joint geometry [3]. In regard to femoral components, this includes the preservation of leg length and an appropriate femoral offset and rotation. As with any arthroplasty, primary stable fixation, enabling proper force transmission to the remaining femoral bone stock, is essential to achieve long-term stability. Whenever it is possible, circumferential press-fit should be applied caudal to the defect situation [4]. Cancellous, as well as cortical defects should be subjected to augmentation to promote biological downsizing and improve the outcome in following revision procedures [5, 6].

A successful revision arthroplasty, therefore, requires decisive preoperative planning and appropriate choice of implant. Common patterns of defect morphology should be evaluated in a consistent and reproducible demeanor resulting in a clear therapeutic algorithm which then can be applied.

Different classification systems have been published to facilitate the aforementioned goals [7–12]. Most of the established classification systems focus on the description of defect location and extent, but fail to relate to current state-of-the-art treatment options. A consensus has not been reached and different systems are being applied in different areas of the world resulting in difficulties when trying to compare the outcome related to defect morphology.

To the authors’ knowledge, no classification system is available that combines intuitive use and structured design while still producing a detailed defect description and offering a therapeutic method according to the current literature and modern state-of-the-art treatment options.

The goal of this publication is to introduce an intuitive guideline for the evaluation and treatment of femoral defects in revision hip arthroplasty. To account for reliability, we compared preoperative grading with intraoperative findings and in the evaluation of reproducibility we evaluated inter- and intra-rater agreement.

We hypothesized that the FDC is a reliable and reproducible classification system. It offers a valid estimation of the defect severity preoperatively and aids with essential planning and preparations. A clear therapeutic algorithm is supplied to help with implant choice and additional interventions according to current literature and expert opinion. The proposed femoral defect classification (FDC) was created to be used alongside the recently introduced acetabular defect classification (ADC). In combination, both defect classification systems offer a structured and holistic approach to hip revision arthroplasty.

## Materials and methods

### Study design and patients

This single center, cohort study is based on retrospectively collected data of patients that underwent femoral revision surgery between 2011 and 2018. A total of 265 consecutive patients who received THA revision for multiple reasons (aseptic loosening, peri-prosthetic infection, recurrent or chronic luxation, implant failure) with exchange of at least the femoral component were identified. Set criteria for exclusion were irretrievable preoperative radiographs or preoperative radiographs of insufficient quality, as well as incomplete intraoperative recordings that did not allow for a reliable grading. Other recorded patient characteristics including age, sex, date of revision and implant inserted were collected. In total, 13 cases were excluded because no digital copies of preoperative radiographs could be retrieved or because radiographs were of insufficient quality. Furthermore, 34 cases were excluded because intraoperative data did not match the defined requirements (intraoperative evaluation). After exclusion, the preoperative X-rays and intraoperative documentation of 218 patients have been included. As the primary outcome parameter, the agreement between preoperative radiographic grading and intraoperative grading has been evaluated using the data of the whole collective (*n* = 218). The evaluation of the rater agreement has been carried out on a randomised sample (*n* = 80) of the whole collective.

### Intraoperative evaluation

Retrospective collection and conversion into FDC grading of intraoperative data were accomplished by the first author. Surgical documentation was analyzed for information concerning the extent of femoral defect morphology and size and combined with additional information such as implants used and/or use of augmentation to allow for proper grading. Cases were excluded if the exact defect location was not specified and if no measurement of defect size was not recorded.

### Radiographic evaluation

To enable blinded assessment, radiographs were striped of any identifying features and anonymized by numerical coding. Therefore, no knowledge of intraoperative findings could distort the grading process. Radiographic grading according to FDC was carried out by the first and last author.

After power analysis according to the formula created by Flack et al., 80 anonymized radiographs were randomly selected and distributed to five raters [13]. The chosen raters are experienced orthopaedic surgeons in the field of hip revision arthroplasty. A teaching session consisting of thorough explanation of the classification system and supervised evaluation of ten random cases was carried out in preparation for the assignment. The 10 sample cases did not include any of the 80 used for later evaluation. A scoring sheet was distributed. None of the raters had any prior knowledge of the FDC or were involved in the creation. To control for distortion due to memory, a wash out period of 2 weeks was established between ratings. Radiographs were re-labelled and randomized prior to the second evaluation.

Preoperative radiographic assessment consisted of standing anteroposterior view and lateral view of the hip joint. Radiographic analysis and grading were carried out using IMPAX EE (Agfa HealthCare GmbH, Bonn, Germany).

### Classification system

The FDC is based on the integrity of the main femoral segments which determine function and structural support. It focuses on the femoral neck, the metaphysis consisting of the greater and lesser trochanter, and the femoral diaphysis. The four main categories determine the location of the defect while subcategories a, b and c are being used to classify the extent of damage in each location.

### Type 1 defects

**Fig. 1:**
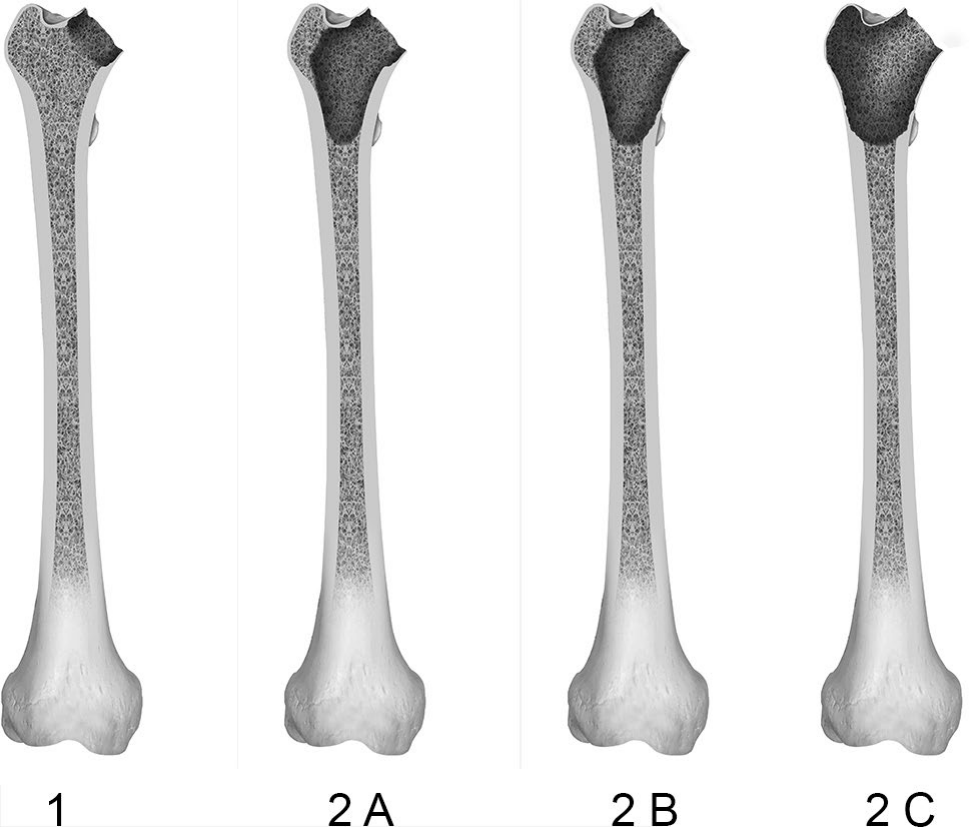
Shows type 1 and type 2 defects of the femoral defect classification (FDC). Type 1 presents a limited defect of the femoral neck; type 2 defects concern the femoral metaphysis with type 2A presenting a cancellous defect, 2B displaying an insufficient calcar femoris and the region of the lesser trochanter and 2C affects the whole metaphysis with calcar femoris, greater and lesser trochanter

Type 1 defects encompass the entire region of the femoral neck while the metaphysis remains uncompromised. Such defects can be encountered during revision surgery of a hip resurfacing prosthesis or in cases of prior osteosynthesis due to femoral neck fractures. An illustration of type 1 is displayed in Fig. 1.

### Type 2 defects

Type 2 defects are limited to the area of the metaphysis. The diaphysial bone remains intact. Type 2 A displays total depletion of the metaphyseal cancellous bone, while the compact bone remains supportive on both the greater and lesser trochanter. In type 2B, the lesser trochanter and the thick compact bone called Calcar femoris are rendered non-supportive in addition to the cancellous depletion described above. In type 2C, severe bone loss and disintegration of the greater trochanter is encountered. This presents a structural as well as functional challenge due to the loss of vital muscle attachment. All type 2 defects are displayed in Fig. 1.

### Type 3 defects

**Fig. 2:**
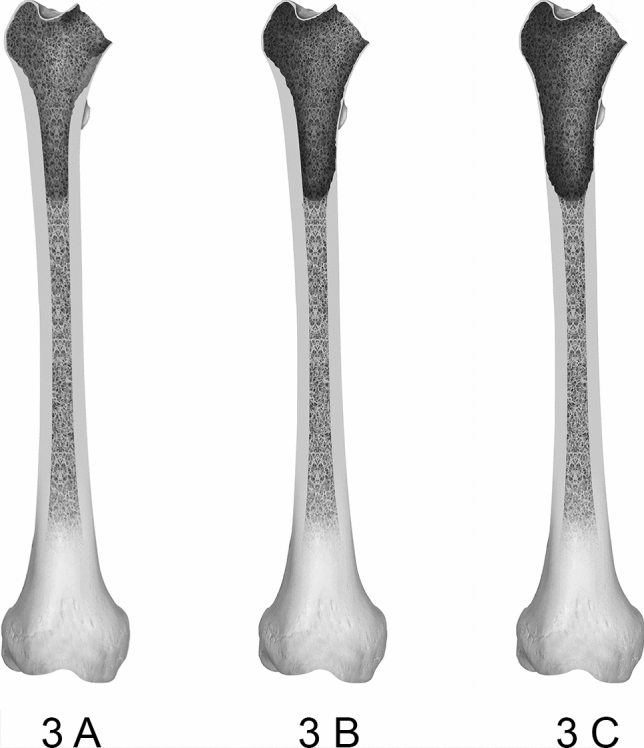
Displays the subdivisions of type 3 defects of the femoral defect classification (FDC). Type 3 defects affected the proximal diaphyseal bone of the femur while leaving enough viable bone stock in the area of the isthmus femoris. The subdivision narrows down defect severity with 3A presenting a full depletion of cancellous bone, 3B displaying an affection of the cortical bone below 50% of the circumference and 3C rendering above 50% of the cortical bone non-supportive

In type 3 defects, the extension of bone loss reaches the diaphysis while respecting the isthmus femoris (region of smallest intramedullary diameter of the femoral diaphysis). Type 3A is defined as a hollowed out cortical bone with complete destruction of the cancellous bone structure. The cortical bone remains to provide circumferential support. In type 3B, in addition to the depletion of cancellous bone, the defected encompasses part of the cortical circumference rendering below 50% of it unsupportive. In type 3C, the defect amounts to > 50% of the cortical circumference. Differences between the individual type 3 defects can be seen in Fig. 2.

### Type 4 defects

**Fig. 3:**
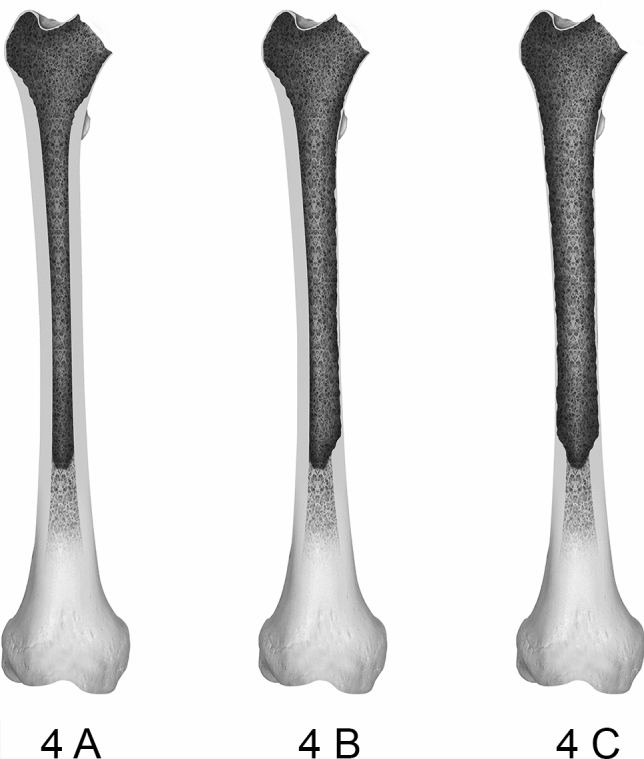
Displays the subdivisions of type 4 defects of the femoral defect classification (FDC). Type 4 defects affected the bone stock of the isthmus femoris presenting the most severe defects encountered in hip revision arthroplasty. Similar to type 2 defects, 4A describes a depletion of cancellous bone, 3B renders up under 50% of the cortical bone of the isthmus femoris non-supportive and for 4C defects over 50% of the cortical bone of the isthmus femoris is compromised

While type 4 defects follow the same structure as type 3 defects, the isthmus femoris is compromised. A graphic representation of type 4 defects is illustrated in Fig. 3.

### Statistical analysis

The statistical analysis was carried out using IBM SPSS Statistics 1.0.0.1131 (IBM Inc., Armonk, New York, USA). The level of significance was set at *p* < 0.05. The confidence interval has been set at 95%. As a means to account for inter-rater reliability in the process of comparing ordered categorical data with more than 2 raters, Fleiss Kappa was used. Through the utilization of Cohens Kappa, intra-rater reliability was calculated and compared through the mean kappa of all raters. Kappa values were interpreted utilizing the agreement scale described by Landis and Koch [14]. Kappa values exceeding 0.80 indicate excellent agreement, between 0.61 and 0.8 indicate good agreement, between 0.41 and 0.60 indicate moderate agreement, between 0.21 and 0.4 indicate fair agreement and between 0.20 and below indicates poor agreement.

## Results

**Fig. 4:**
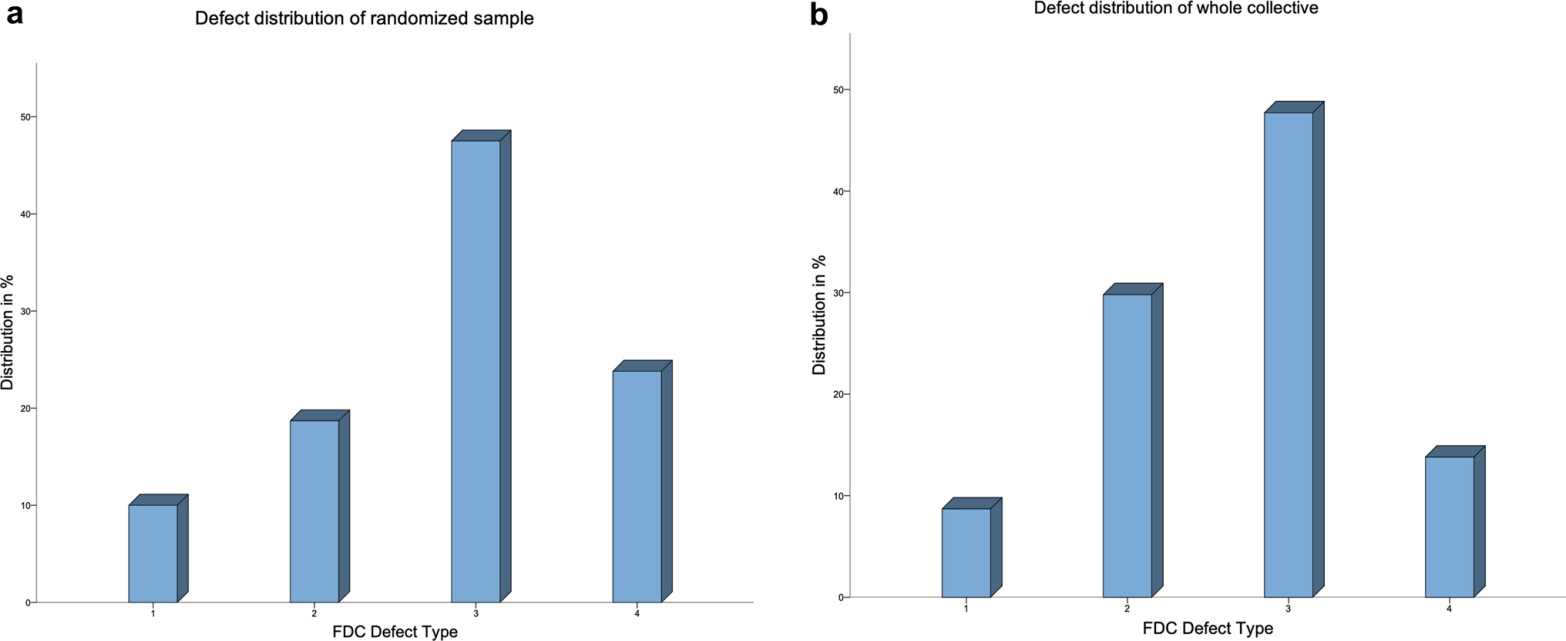
To allow for proper evaluation of reliability each FDC defect type is in included in the whole collective, as well as in the randomized sample and the distribution is considered to be similar to the occurrence in the clinical practice of femoral revision arthroplasty. The whole collective consisted of 218 cases: 19 Type 1 defects 65, Type 2 defects (2A *n* = 37, 2B *n* = 1, 2C *n* = 27) 103, Type 3 defects (3A *n* = 72, 3B *n* = 15, 3C *n* = 16), 31 Type 4 defects (4A *n* = 17, 4B *n* = 3, 4C *n* = 11), (**a**) Illustration of the distribution of FDC defect types in the whole collective (*n* = 218); y-axis: percent out of all cases, x-axis: FDC Defect type 1–4; (**b**) Illustration of the distribution of FDC defect types in the randomized sample (*n* = 80); y-axis: percent out of all cases, x-axis: FDC Defect type 1–4

A total of 218 cases have been included to evaluate the agreement between preoperative radiographic grading and intraoperative grading. Indications for revision were aseptic loosening (*n* = 153), peri-prosthetic infection (*n* = 47), recurrent or chronic luxation (n = 15), implant failure *n* = 3). Preoperative radiographs as well as intraoperative findings have been graded according to FDC. Mean interval between preoperative X-ray and intraoperative assessment was 12.7 ± 13.4 days. According to the intraoperative gradings, the samples proved to be well-balanced and exhibited every defect type. The defect distribution of the whole population and of the randomized sample are displayed in Fig. 4.

A Cohens kappa of 0.832 ± 0.028 could be evaluated, accounting for excellent agreement between preoperative radiographs and intraoperative findings.

**Fig. 5:**
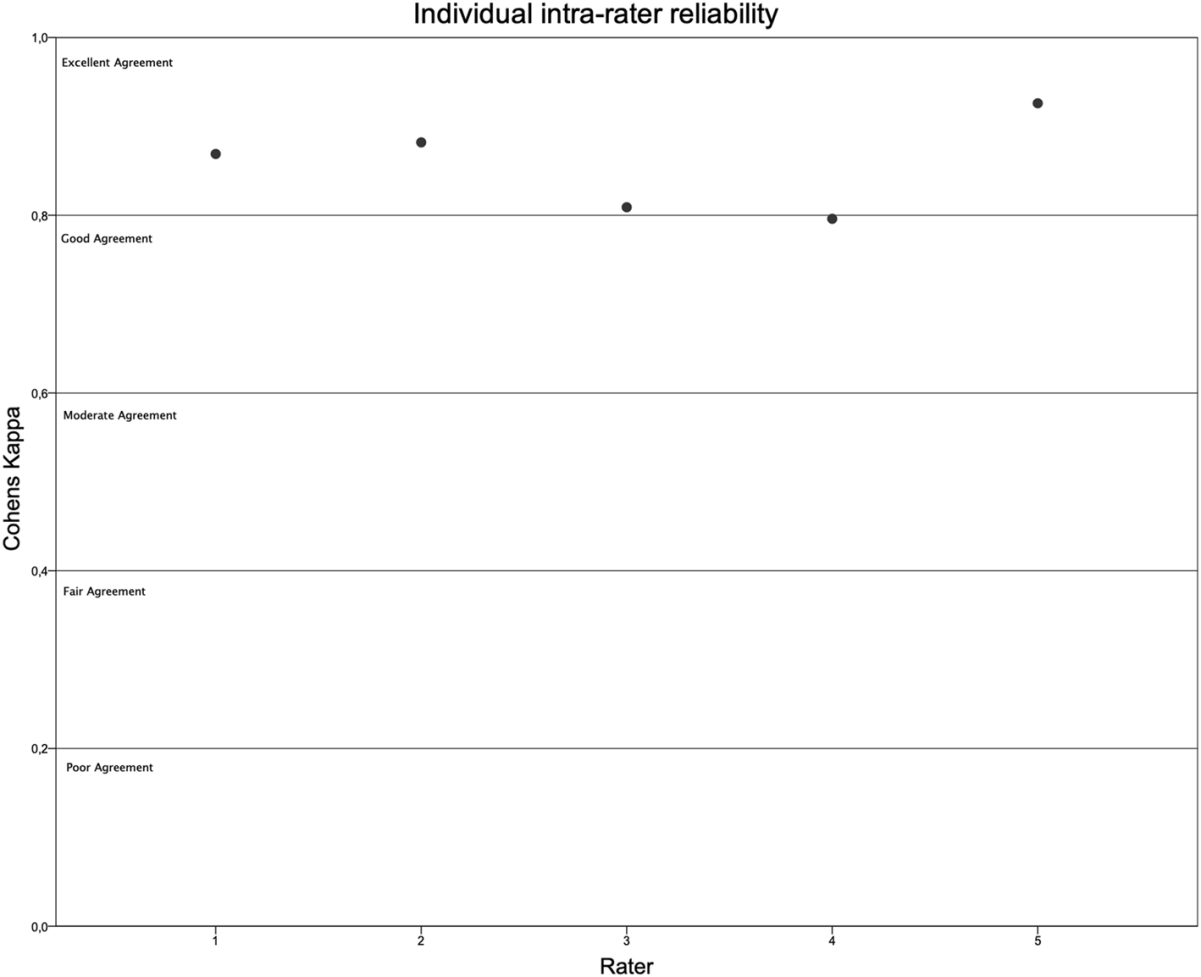
Illustration of individual intra-rater reliability. All individual raters displayed good to excellent agreement between themselves at different time points. y-axis: Cohens Kappa, x-axis: individual rater

To account for inter-rater reliability, a randomised sample of 80 patients have been evaluated by five different observers. Testing for inter-rater reliability, a Fleiss Kappa of 0.688 (low CI 0.660; high CI 0.716) could be evaluated falling into the good agreement range as defined by Landis and Koch [14]. When testing for intra-rater reliability Cohens Kappa of each of the five raters has been analyzed and the mean was evaluated at 0.856 ± 0.054 accounting for excellent agreement according to Landis and Koch [14]. Individual results for each rater are displayed in Fig. 5.

## Discussion

The most important feature of a classification system is a clinical usefulness generated through a clear therapeutic algorithm. The loosening of the femoral component of a hip prosthesis is commonly associated with peri-prosthetic bone loss which can hinder primary stable fixation. Reliable defect recognition and classification is essential for successful femoral revision arthroplasty. Depending on defect location and morphology, different treatment options should be applied.

So far numerous classification systems have been introduced to rate femoral bone defects. The AAOS introduced the most extensive one in 1993 describing the full range of femoral abnormalities found in primary and revision hip arthroplasty. This approach resulted in a rather bulky but comprehensive classification system which, however, fails to offer a clear therapeutic guidance [8, 10]. Gross et al. focused primarily on bone allograft augmentation to enable reconstruction and primary stable implantation [10, 11]. Other classification systems mainly cater to a specific therapeutic approach. The Endoklinik’s system has been constructed to evaluate the option of cemented revision arthroplasty while Engh and Paprosky aimed for primary stable, diaphyseal implantation of a cementless stem [7, 10, 12]. To improve upon the existing approaches, the FDC takes augmentation, all state-of-the-art implant choices and defect morphology into account, to offer a complete, yet intuitive classification system. The FDC was created to complete the ADC and in union, they offer a holistic approach to hip revision arthroplasty [2]. It is a reliable, reproducible and intuitive classification system which, in addition, offers a clear therapeutic guideline according to state-of-the-art treatment options.

Presenting the same structure as the familiar ADC, it can be applied to native radiographs of the femur. Therefore, it is applicable to a widely available and easily reproducible diagnostic process. In the present analysis the extent of the defect is estimated in preoperative radiographs when compared to intraoperative findings show a *k* value of 0.83. As defined by Landis and Koch, this indicates excellent agreement [14]. The authors, therefore, conclude that the FDC presents a reliable option to evaluate defect severity preoperatively. As an example, for an esthablished classification the Paprosky system has been evaluated for validity twice. Gozzard et al. reported moderate agreement between preoperative radiographical grading and intraoperative grading (*k* = 0.54) [15]. The group around Käfer confirmed these findings with *k* values from 0.59 to 0.68 [16]. These results are in contrast to our own excellent agreement, which might be partially contributed to improvements in imaging technique since the conduct of the studies from Gozzard and Käfer and to the structured assessment the FDC provides.

This is especially important for the practitioner to confidently approach a revision case. The good results might be partially contributed to the structured radiographic analysis aided by the provided evaluation spreadsheet and standardized defect definitions according to easily observable radiographic landmarks. In this study, standing anteroposterior view and lateral view X-rays of the affected hip have been evaluated. Due to cost- and practicability-related causes, x-rays of the femur still present the method of choice for most orthopaedic surgeons to monitor bone stock and other stability parameters after primary or revision arthroplasty in the postoperative follow up cycle [17]. A classification system should be applicable to the most common imaging. It is important to mention that radiopaque material can severely impeded defect recognition and assessment of defect severity. In addition, the extent of damage to the bone caused by the revision operation due to implant and/or cement removal is difficult to anticipate and can significantly change the defect type. These points need to be taken into consideration when planning for a revision operation. A wide portfolio of available implants, especially revision stems, is advisable. Advanced diagnostics can be applied for specific questions. Through considerable improvements in diagnostic software and hardware, MARS (metal artefact reduction sequence) MRI has recently been shown to present good sensitivity and specificity to detect component loosening [18]. CT scans offer improved resolution and are useful in the analysis of focal osteolysis, deformities such as rotational abnormalities and improved digital planning capacities [19–21]. X-ray still present the first imaging step when loosening of hip prosthesis is suspected and present with a decent sensitivity and specificity but are not as specific/sensitive as MRI and CT [22].

For a classification system to be widely adopted, it needs to be intuitive and easy to apply. In the creation of the FDC, the authors focused on a structured design and kept it as similar as possible to the ADC to complete the integrated system.

Several studies have evaluated the reliability of established femoral bone loss classifications. The most evaluated system is the Paprosky classification with mixed results in literature. An evaluation carried out by the same group who introduced the initial classification system found substantial agreement between raters and for the same rater at different points in time. Results showed *k* values of 0.61 for inter-rater reliability and 0.81, 0.78, and 0.75 for intra-rater reliability which is in a similar range to our own findings [23]. These results were confirmed by another study, which also evaluated the classifications system of the AAOS (0.63–0.68) and the Endoklinik (0.83–0.85) [24]. Other groups have reported different results with reliability rating as low as 0.27–0.50 for inter-rater evaluation and 0.09–0.64 for intra-rater evaluation of the Paprosky system [15]. Haddad et al. described an overall low reliability for the Paprosky, AAOS and Mallory system with inter-rater reliability ranging from 0.12 to 0.29 and intra-rater reliability ranging from 0.43 to 0.63 [25]. A recent systematic review demonstrated good intra-rater reliability for the Paprosky and AAOS systems but only moderate inter-rater-reliability for Paprosky and even poor inter-rater reliability for AAOS [26]. An explanation for the deviating results in different studies might be differences in training [23]. In summary, the FDC appears to be in an equal or even superior range concerning reliability when compared to established classification systems. Due to different results in the evaluation of reliability of established classification systems further independent studies may be helpful to further validated the FDC.

Similar studies have been conducted with acetabular defect classification systems and the results for rater reliability fall into a similar range [27]. A direct comparison is limited.

In the following paragraphs, the therapeutic recommendations according to defect type will be discussed. The therapeutic algorithm was established through expert opinion in combination with a thorough review of the current literature and derived from the therapeutic procedure of the authors’ own clinical experiences.

The category of type 1 bone defects presents with a destruction of the femoral neck while leaving the remaining bone stock of the femur, including the metaphysis and diaphysis, utterly intact. These defects can be experienced after a femoral neck fracture or a revision of a hip resurfacing prosthesis. While extensive research shows the higher failure rate of metal-on-metal (MoM) hip resurfacing when compared to a standard total hip replacement, a limited number of studies investigated the revision implant best suited to the consecutive femoral bone defect after implant failure. In most publications, a stable femoral revision after hip resurfacing could be achieved using a short stem or a standard cemented/cementless femoral component [28, 29].

The main defect for FDC type 2 is located at the metaphysis. Type 2A presents a complete depletion of the metaphyseal cancellous bone, while the structure of the compacta remains supportive, leaving especially the muscle attachment at the greater and lesser trochanter intact. This type of defect can occur after extensive loosening of a short stem prosthesis or a cementless or cemented standard stem. As long as the cortical bone stock of the metaphysis is supportive, a metaphyseal anchoring standard stem can be utilized [30–33]. When a cemented fixation is chosen, enough cancellous bone, or at least a roughened up corticalis needs to remain in the diaphyseal aspect to enable proper interdigitation with the cement layer [34]. In case of cementless stem fixation, impaction bone grafting should be applied to allow for biological downsizing of the metaphyseal defect [35–39].

In type 2B, the lesser trochanter and the dense compacta called Calcar femoris are rendered non-supportively in addition to the cancellous depletion of the metaphyseal aspect of the femur. This renders the metaphyseal bone stock non-supportively and dictates to project the fixation of the chosen prosthesis in the diaphyseal portion of the femur. Due to the intact greater trochanter and the muscle attachment of the gluteal group, a cementless or cemented diaphyseal anchoring standard stem can be chosen. Once the stem is inserted, analogous to type 2A defects, impaction bone grafting should be applied to allow for biological downsizing [35–37].

**Fig. 6:**
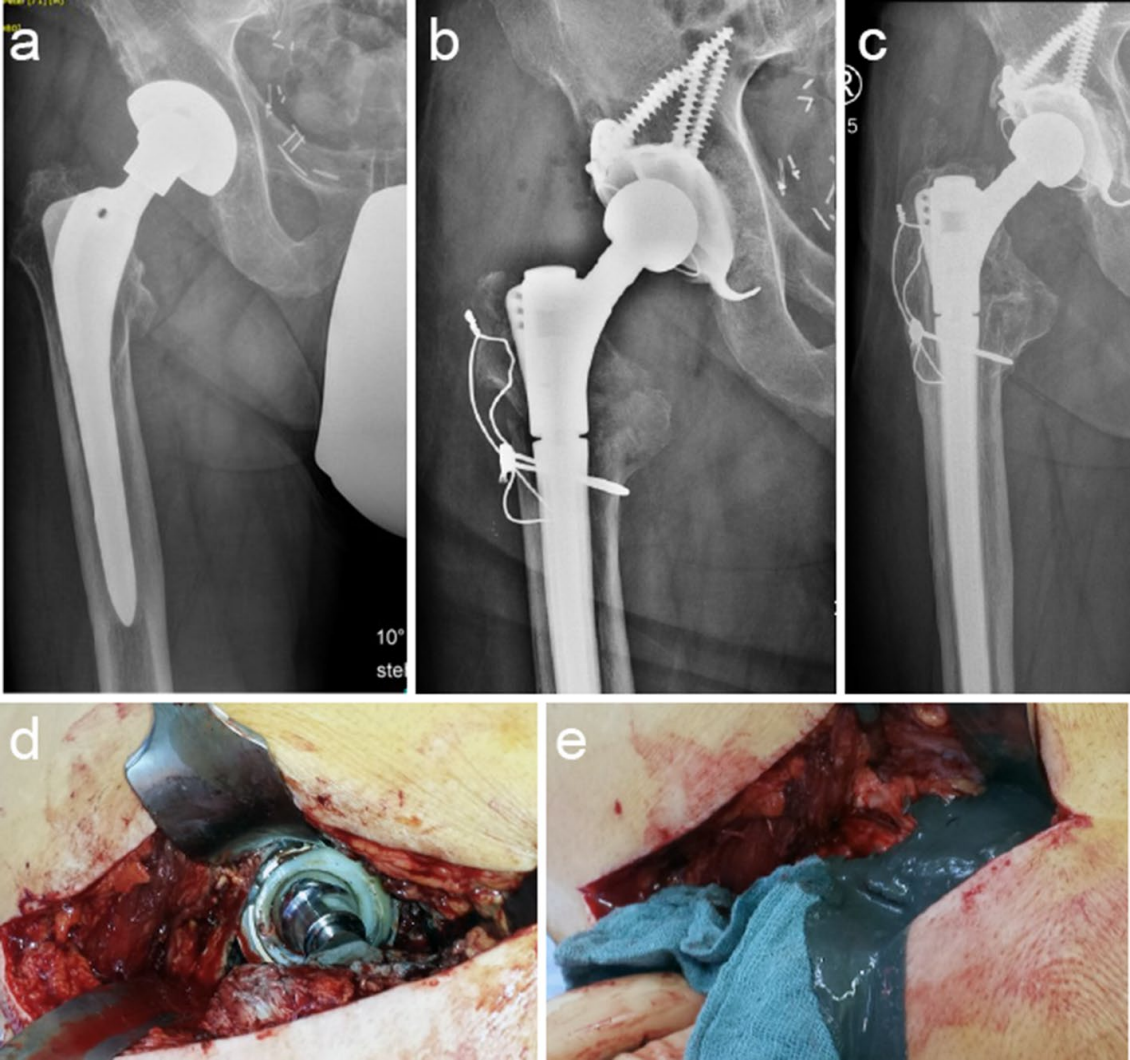
Clinical case of a 73-year-old, male patient presenting with a type 2C defect caused by severe metallosis due to metal-on-metal particle debris. Primary arthroplasty took place 12 years prior to revision; (**a**) displays the preoperative native radiograph (pelvis ap standing) with osteolytic bone stock at the metaphysis; (**b**) postoperative native radiograph (pelvis ap standing) with a cementless, modular, femoral revision stem and additional wire cerclages to stabilize the remaining bone stock of the greater trochanter combined with impaction bone grafting; (**c**) native radiograph (pelvis ap standing) four years after revision arthroplasty displaying a consolidation of the augmented and stabilized femoral metaphysis; (**d**/**e**) displays the intraoperative finding of severe metallosis

In type 2C, severe metaphyseal bone loss and disintegration of the greater trochanter is encountered. This presents a structural as well as functional challenge due to the loss of vital muscle attachment. Great care should be taken intraoperatively to re-fixate any remaining bone stock and muscle attachments through wire/suture cerclages. To prevent particle debris and consecutive osteolysis through metallosis, the wire/suture cerclages should not be in contact with any part of the endoprosthesis. To ensure an appropriate intermediate layer, Strut grafts may be applied. Figure 6 demonstrates a type 2C defect caused by severe metallosis for which the deficient bone stock of the greater trochanter is stabilized with wire/suture cerclages.

Caused by the decreased function of the muscular sleeve, instability with the risk of consecutive luxation increases dramatically. While fixation can be handled according to type 2B with a diaphyseal anchoring stem, which can either be cemented or cementless, additional support to provide functional stability needs to be considered. Alternative bearing constructs can include dual mobility articulations, unconstrained tripolar articulations or constrained liners. While constrained liners offer an increased amount of stability, they further the risk of potential catastrophic implant failure requiring additional surgical intervention [40, 41]. Therefore, the addition of a constrained liner should be reserved for severe cases with previous exhaustion of other options. Unconstrained tripolar articulations and dual mobility articulations present less constrained options, while still offering a larger excursion distance, more range of motion prior to impingement and can help in the restoration of appropriate offset and the tensioning of the abductor muscle. First results show promising results for tripolar and dual mobility articulations regarding rate of luxation. The increased number of articulating interfaces may lead to an increased amount of particle debris and consecutive osteolysis, which raises concerns [42–46].

**Fig. 7:**
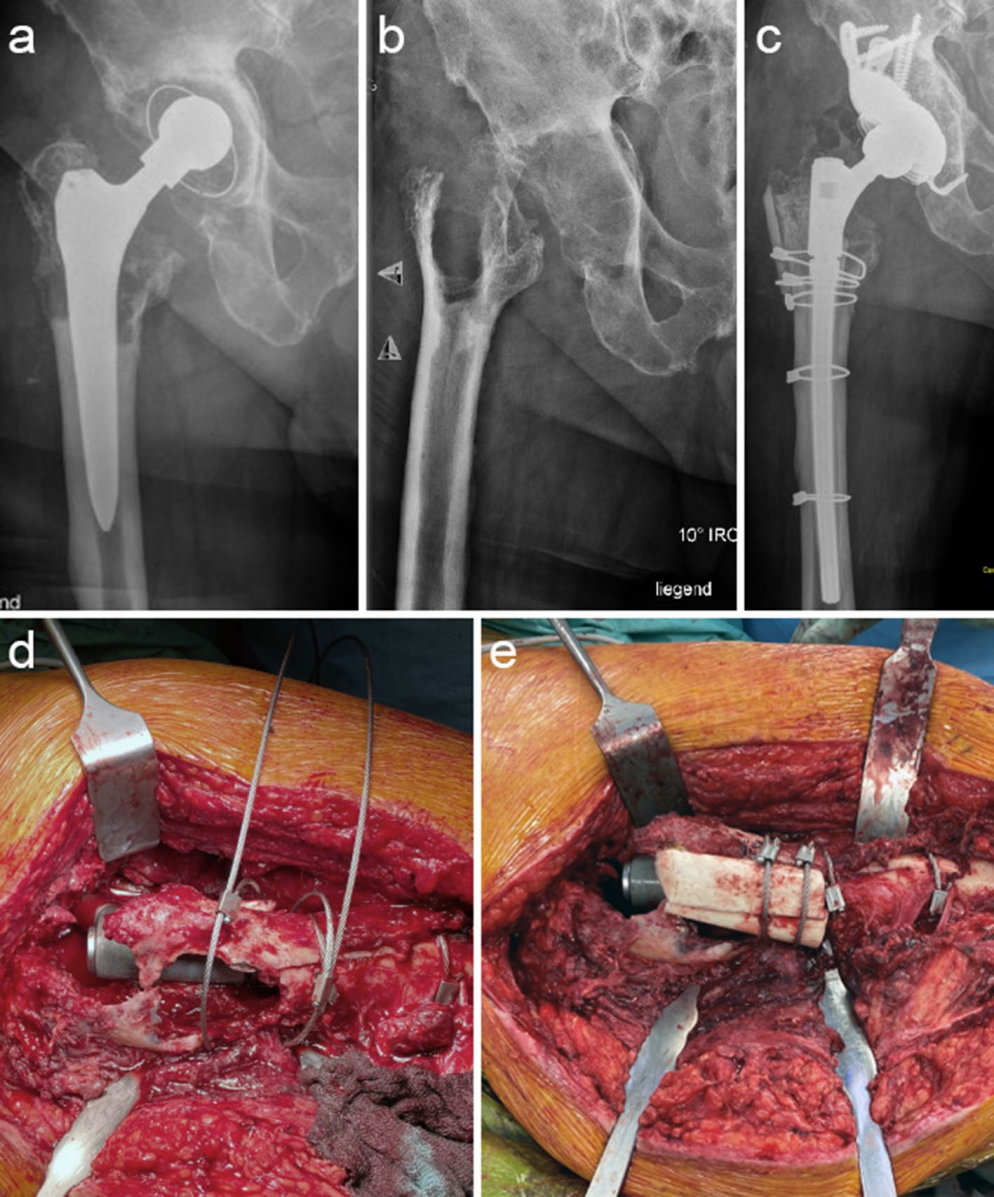
Clinical case of a 71-year-old, male patient presenting with a type 3C defect and cystic granulomatosis due to particle debris. **a** displays the preoperative native radiograph (pelvis ap standing), which illustrates large cystic osteolysis encompassing the metaphysis and proximal diaphyseal bone; (**b**) postoperative native radiograph (pelvis ap standing) after removing the implant and granuloma visualizing the full extent of the defect situation; (**c**) postoperative native radiograph (pelvis ap standing) after revision arthroplasty utilizing a long, cementless, modular, femoral revision stem and additional allogenous augmentation through impaction bone grafting and Strut grafts fixated with wire cerclages; (**d**) illustrates the extent of the metaphyseal/diaphyseal defect, the stem has been inserted and subsequently wire cerclages are being placed around the femur to receive the Strut grafts; (**e**) finalized femoral revision arthroplasty. Strut grafts act as a shell for the impaction bone grafting

Type 3 defects extend into the diaphyseal aspect of the femur while leaving the isthmus femoris intact. In these cases, a cementless revision stem (modular/monobloc) should be implanted with a sufficient length to anchor in the cortical frame distal to the defect. The required length of circumferential press-fit for cementless revision stems is an ongoing debate. A press-fit distance for tapered stems of less than 2 cm appears to be an independent risk factor for significant stem subsidence [45]. Other authors reported a minimum of 5–7 cm of circumferential press-fit required to prevent subsidence, which appears adequate in our own clinical experience. Drilling of the femoral medullary canal can be utilized to optimize fitting of the stem and to achieve the appropriate contact area [38, 46]. To neutralize applied endo-femoral force generated through the press-fit, wire/suture cerclages can be liberally applied. To allow for biological downsizing, especially in type 3C defects, Strut grafts can be added in the area of the unsupportive bone to provide a framework for wire/suture cerclage stabilized diaphyseal cortical bone. In Fig. 7, Strut grafts are applied to provide additional augmentation in the case of a type 3C defect. If a sufficient press-fit of a minimum of 5 cm cannot be achieved, additional distal locking screws should be added to the construct.

Finally, type 4 defects present the most severe defects of this classification system and in clinical practice. The isthmus becomes increasingly compromised while moving from a to c. Type 4A and B defects may be able to be salvaged through long cementless revision stems and a combination of Strut grafts and wire/suture cerclages to enable at least a limited amount of press-fit. Type 4C defects display the so called “stovepipe configuration” with an insufficient isthmus femoris. In these cases, cemented partial or total femur replacement should be applied as a salvage procedure. A table containing all therapeutic recommendations according to defect type is supplied below (Table 1).

**Table 1:** Therapeutic recommendation based on defect type according to FDC

Type of defect	Implant choice
1	Cementless or cemented standard stem, short stem if enough of the femoral neck remains
2A	Cementless short stem, cementless or cemented standard stem
2B	Diaphyseal anchoring stem design + impaction bone grafting
2C	Diaphyseal anchoring stem design + impaction bone grafting + alternative bearing construct*
3A	(Modular) Revision stem + impaction bone grafting
3B	(Modular) Revision stem + impaction bone grafting + as an option: diaphyseal wire/suture cerclages
3C	(Modular) Revision stem + impaction bone grafting + strut graft proximal lateral, stabilized with wire/suture cerclages; as an option: diaphyseal wire cerclages
4A	(Modular) Revision stem + impaction bone grafting + strut graft and wire/suture cerclages
4B	(Modular) Revision stem with distal screw fixation + impaction bone grafting + strut graft and wire/suture cerclages
4C	Proximal or total femoral replacement

*Whenever insufficient muscular stability is encountered, alternative bearing constructs (bipolar/tripolar) may be applied

This classification system has several limitations. To keep the structure simple and easy to apply, some defect morphologies are simplified. A standardized classification system will always lack in the all-encompassing description of every possible defect morphology. In clinical practice, different combinations of defects might be encountered and the surgeon must be vigilant to alter the therapeutic algorithm accordingly. While the FDC is applicable to any type of radiographic imaging as well as to an intraoperative setting, it has initially been based on x-rays. As discussed above, x-rays may present with limited reliability in defect recognition. When performing a preoperative evaluation with an implant still in situ, the prospective additional destruction of bone due to implant removal must be taken into consideration and the defect grading must be updated accordingly. The correlation between preoperative grading and intraoperative findings has been evaluated retrospectively. While further prospective studies are ongoing, the current evaluation presents with limited expressiveness. The preoperative grading and the intraoperative findings have been evaluated by two of the originators of the FDC which can propose a possible bias.

## Conclusion

The femoral defect classification (FDC) is a reliable and reproducible classification system and in combination with the recently introduced acetabular defect classification (ADC) offers a structured approach to the entire field of hip revision arthroplasty. The provided therapeutic algorithm has been created according to current literature and expert opinion and should be validated through future prospective clincial research.
